# Successful management of the delayed leaflet perforation after transcatheter edge-to-edge repair procedure using transcatheter occlusion

**DOI:** 10.1093/ehjcr/ytae103

**Published:** 2024-02-20

**Authors:** Da Zhu, Chun-mei Xie, Shou-zheng Wang, Xiang-bin Pan

**Affiliations:** Department of Structure Heart Center, Chinese Academy of Medical Sciences, Fuwai Yunnan Hospital, Affiliated Cardiovascular Hospital of Kunming Medical University, 528 Shahebei Road, Wuhua District, Kunming 650102, China; Department of Anesthesiology, Chinese Academy of Medical Sciences, Fuwai Yunnan Hospital, Affiliated Cardiovascular Hospital of Kunming Medical University, 528 Shahebei Road, Wuhua District, Kunming, China; Department of Structure Heart Center, Chinese Academy of Medical Sciences, Fuwai Yunnan Hospital, Affiliated Cardiovascular Hospital of Kunming Medical University, 528 Shahebei Road, Wuhua District, Kunming 650102, China; Department of Structure Heart Center, Fuwai Hospital, National Center for Cardiovascular Diseases, Chinese Academy of Medical Sciences and Peking Union Medical College, 167 Beilishi Road, Xicheng District, Beijing 10B0037, China; Department of Structure Heart Center, Chinese Academy of Medical Sciences, Fuwai Yunnan Hospital, Affiliated Cardiovascular Hospital of Kunming Medical University, 528 Shahebei Road, Wuhua District, Kunming 650102, China; Department of Structure Heart Center, Fuwai Hospital, National Center for Cardiovascular Diseases, Chinese Academy of Medical Sciences and Peking Union Medical College, 167 Beilishi Road, Xicheng District, Beijing 10B0037, China

## Case description

**Figure 1:**
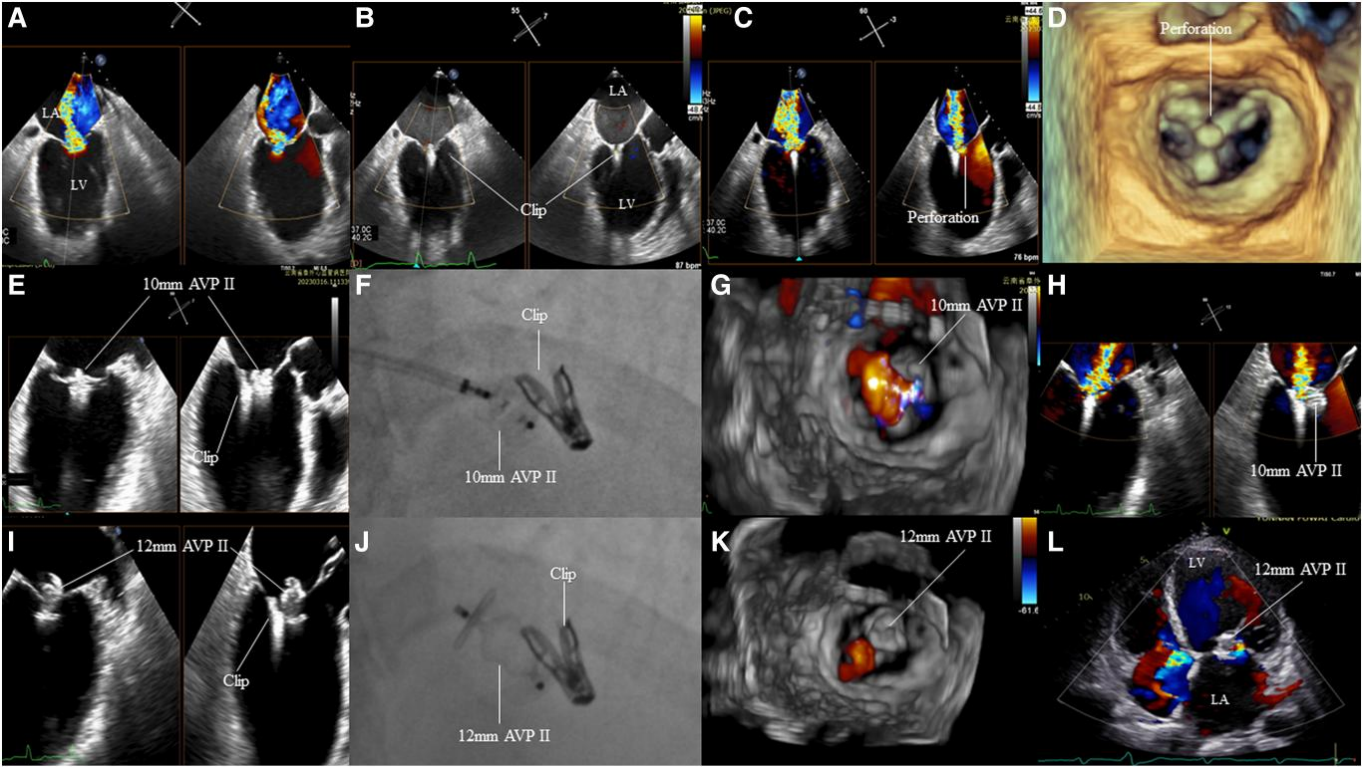
(*A*–*D*) Recurrent mitral regurgitation (MR) was noted due to anterior leaflet tear. (*E*–*H*) Suboptimal MR reduction and occluder morphology were noted due to insufficient oversize [10 mm Amplatzer Vascular Plug (AVP) device]. (*I*–*K*) After using 12 mm AVP with sufficient oversize, MR was reduced to 1+ to 2+. (*L*) Transoesophageal echocardiography in 1-year follow-up revealed 1+ MR.

A 55-year-old man was diagnosed with dilated cardiomyopathy, chronic heart failure, paroxysmal atrial fibrillation, and severe non-ischaemic functional mitral regurgitation (MR) despite optimal medical treatment. Echocardiogram revealed 4+ MR with symmetrical leaflet tethering. Ejection fraction was 26% with left ventricular end-diastolic diameter 70 mm. Coaptation height was 2.0 mm with vena contracta width 10 mm (*Figure 1A*). Mitral valve area was 5.4 cm^2^ with mean trans-valvular gradient 1 mmHg. Transcatheter mitral edge-to-edge repair (TEER) was recommended.

Transcatheter mitral edge-to-edge repair was done under the guidance of transoesophageal echocardiography (TEE). A single clip was implanted in a central A2/P2 position, reducing the MR from severe 4+ to mild 1+ (*Figure 1B*). Tension was loosened during clip closure. Mean left atrial pressure decreased from 30 to 15 mmHg after the procedure. The patient was discharged 9 days after procedure, and post-operative period was uneventful. One month after TEER, echocardiography revealed recurrent 4+ MR due to anterior leaflet perforation in the A2 region, with a defect diameter of about 5 mm (*Figure 1C* and *D*). Considering the high risk of open-heart surgery, transcatheter occlusion was attempted. A 14F steerable catheter was placed in the left atrium through the previous puncture site, and the guide wire was placed into the left ventricle through the tear hole. First, we chose a 10 mm Amplatzer Vascular Plug (AVP) II (St. Jude Medical, Abbott), and TEE revealed suboptimal MR reduction and occluder morphology due to insufficient oversize (*Figure 1E–H*) according to our previous experience.^1^ Therefore, the 12 mm AVP II was used, and the waist of the occluder was fully fitted into the perforation hole with sufficient oversize. Mitral regurgitation was reduced to 1+ to 2+ after deployment (*Figure 1I–K*). Post-operative recovery was uneventful, with no sign of haemolysis. In the 1-year follow-up, the patient presented good condition [New York Heart Association (NYHA) class 2] with 1+ MR (*Figure 1L*), and both the clip and the plug were in a stable position.

**Consent:** The authors confirm that written consent for publication of this case report was obtained from the patient in line with the Committee on Publication Ethics (COPE) guidance.

**Funding**: This work was supported by the National Key Research and Development Program (2022YFC2503400), the Yunnan Provincial Clinical Research Center for Cardiovascular Diseases (202102AA310002), and the Major Science and Technology Special Plan Project of Yunnan Province (202302AA310045).

## Data Availability

Data are available on request.
